# Enhancement of *Aedes albopictus* collections by ovitrap and sticky adult trap

**DOI:** 10.1186/s13071-016-1501-x

**Published:** 2016-04-21

**Authors:** Enkelejda Velo, Perparim Kadriaj, Kujtim Mersini, Ada Shukullari, Blerta Manxhari, Artan Simaku, Adrian Hoxha, Beniamino Caputo, Luca Bolzoni, Roberto Rosà, Silvia Bino, Paul Reiter, Alessandra della Torre

**Affiliations:** Control of Infectious Diseases Department, Institute of Public Health, Tirana, Albania; National Veterinary Epidemiology Unit, Food Safety and Veterinary Institute, Tirana, Albania; Department of Biology, Faculty of Natural Sciences, Tirana, Albania; Dipartimento di Sanità Pubblica e Malattie Infettive, Università di Roma “Sapienza”, Rome, Italy; Istituto Zooprofilattico Sperimentale della Lombardia e dell’Emilia Romagna, Parma, Italy; Dipartimento di Biodiversità ed Ecologia Molecolare, Centro Ricerca e Innovazione, Fondazione Edmund Mach, San Michele all’Adige, Trento, Italy; Insects and Infectious Disease Unit, Institute Pasteur, Paris, France

**Keywords:** Mosquito monitoring, Mosquito surveillance, *Aedes*, Ovitrap, Sticky trap, Hay-infusion, Albania, Europe

## Abstract

**Background:**

In the last decades, *Aedes albopictus* has become an increasing public health threat in tropical as well as in more recently invaded temperate areas due to its capacity to transmit several human arboviruses, among which Dengue, Chikungunya and Zika. Enhancing the efficiency of currently used collection approaches, such as ovitraps and sticky traps, is desirable for optimal monitoring of the species abundance, for assessment of the risk of arbovirus transmission and for the optimisation of control activities.

**Findings:**

Two sets of 4 × 4 Latin-square experiments were carried out in Tirana (Albania) to test whether modifications in ovitrap shape and size and in oviposition substrate would increase collections of *Ae. albopictus* eggs and whether hay-infusion would increase adult catches by sticky trap. Generalized Linear Mixed Models with negative binomial error distribution were carried out to analyse the data. Cylindrical ovitraps lined with germination paper yielded significantly higher egg catches than those exploiting either the (commonly used) wooden paddles or floating polystyrene blocks as oviposition substrates. No difference was observed between cylindrical and conical shaped ovitraps. Ovitraps and sticky traps baited with hay infusion yielded significantly higher egg and adult catches than un-baited ones. A significant relationship between ovitrap and sticky trap catches was observed both in the absence and in the presence of attractants, with ovitrap catches increasing more than sticky trap catches at increasing adult female densities.

**Conclusions:**

This study provides grounds for optimisation of ovitraps and sticky traps as monitoring tools for *Ae. albopictus* by (i) supporting use of germination paper as most appropriate oviposition substrate; (ii) suggesting the possible use of stackable conical ovitraps for large scale monitoring; (iii) confirming the use of hay-infusion to increase egg catches in ovitraps, and showing that hay-infusion also significant increases adult catches by sticky traps.

## Background

*Aedes albopictus* is the mosquito species which has been capable of the widest geographical expansion thus becoming a public health threat in tropical as well as in more recently invaded temperate areas due to its capacity to transmit several human arboviruses, among which Dengue, Chikungunya and Zika [1, 2].

Surveillance and monitoring of the species is thus an instrumental activity to be carried out to prevent infestation of new areas, to assess the risk of arbovirus transmission and to optimize control activities. Ovitrap is the simplest and most widely used monitoring device for *Ae. albopictus*, as well as of other container-breeding species such as *Aedes aegypti*, the major vector of yellow fever, dengue and Zika [3]. Ovitrap is a small black plastic vessel mimicking the preferred breeding site for the species, i.e., tree-holes, rock-holes and other small natural containers in its original habitat in south-east Asia, and small man-made containers in more recently colonized urban environments [4]. The vessel is partly filled with water and either contains a wood or masonite rough paddle standing in the water or has the internal walls lined with seed germination paper for females to lay eggs. Mosquito presence and abundance is indirectly estimated by counting eggs laid on the paddle or on the germination paper. Baiting ovitrap with hay-infusion has been shown to increase *Ae. albopictus* egg catches [5–11]. A more direct approach to monitor the same fraction of *Ae. albopictus* population monitored by ovitrap (i.e. oviposting females) is represented by sticky traps, which basically are ovitraps whose internal walls, or some internal additional structures, are lined with adhesive films to which the mosquitoes approaching the traps remained stuck [12, 13].

The aims of this study were to test under field conditions whether (i) some small modifications in shape and size and oviposition substrate could increase *Ae. albopictus* ovitrap-catches; (ii) hay-infusion could increase sticky trap catches, as already shown for ovitraps; and (iii) ovitrap and sticky traps baited with hay-infusion maintain the correlation shown to occur in the absence of the infusion [13].

## Methods

### Collection methods

Ovitraps (Ov) and Sticky traps (ST [13]) were used to collect *Ae. albopictus* eggs and adults, respectively. Two shapes of black plastic ovitrap were tested: Ov-A, a cylindrical vessel, 9 cm high, 11 cm in diameter with an overflow hole at 7 cm from the base, and Ov-B, a truncated cone (12 cm high; 6 cm lower diameter; 8 cm upper diameter; overflow hole at 9 cm from the base). Three types of oviposition substrates were provided to Ov-A: heavy-weight seed germination paper lining the internal walls (Ov-A1); a floating block of white polystyrene (5 × 5 × 2.5 cm; Ov-A2), and a wooden paddle (12.5 × 2.5 cm) with one rough side (Ov-A3 and Ov-B).

### Experiment 1

Oviposition rates in Ov-A1, Ov-A2, Ov-A3 and Ov-B were compared in 4 × 4 Latin-square experiments carried for 20 weeks from June to October 2011 in nine suburban sites (located at > 500 m from each other) within a 2.25 ha area in Tirana in Albania, the first country in a Europe to be infested [14]. In each experimental site, the four ovitrap types were located in shaded sites at the corners of a 50 m-square area and rotated clockwise on a weekly basis, so that each trap was in the same position every four weeks. Egg counting was carried out under a stereomicroscope in the lab.

### Experiment 2

The collection capacity of Ov-A1 and ST either baited or not with hay-infusion (60 g hay in 10 l of water fermented in open buckets at room temperature for one week) was assessed by Latin-square experiments carried out in July-August 2011 in 20 suburban sites in Tirana (located at > 500 m from the each other). In each site, the four traps (Ov-A1 and ST with clean water and Ov-A1 and ST with hay-infusion) were located in shaded positions at the corners of a 50 m-square area and rotated clockwise on a weekly basis for four weeks. Eggs were counted as in Experiment 1. Adults collected by sticky traps were counted and morphologically identified [15].

### Statistical analysis

Generalized Linear Mixed Models (GLMM) with negative binomial error distribution were carried out to analyse the data. For Experiment 1, the model response variable were egg catches and the explanatory variable was ovitrap type. For Experiment 2, the model response variables were egg and adult female catches for Ov-A1 and ST, respectively, while the explanatory variable was presence/absence of hay-infusion (treatment). In both GLMMs julian day of collection and site were included as random crossed effects. Since likelihood ratio test (LRT) carried out to compare models either including or not the random effects showed that the role of crossed random effects was highly significant (*P*-value of LRT < 10^−6^), these were included in the best models for both experiments.

Correlations between Ov-A1 and ST weekly catches and between averaged catches over the four week-long Experiment 2 were assessed by Kendall’s rank test. Moreover, after checking for the normality of error distribution (Shapiro-Wilkoxon normality tests, Ov-A1: W = 0.962, *P* = 0.201; ST: W = 0.988, *P* = 0.949), a standardized major axis regression (SMA) was used as in [13] to assess the relationship between means of log-transformed catches of Ov-A1 and ST in each site over the four weeks and to test whether this relationship was affected by hay-infusion. Specifically, SMA was preferred to a classical linear regression since sampling errors were expected to occur both for Ov-A1 and ST trap catches [16]. Analyses were carried out using R version 3.2.0, with “glmmADMB” and “smatr” packages.

## Results and discussion

Overall, 104,143 *Ae. albopictus* eggs were collected in Experiment 1 and 19,541 eggs, 830 adult females and 71 males were collected in Experiment 2 (Table 1). No adult specimens belonging to other *Aedes* spp. were collected, confirming the exclusive presence of *Ae. albopictus* as a container-breeding mosquito in the area.

**Table 1:** Mean number ± standard deviation of *Aedes albopictus* eggs/ovitrap/day and adult females/sticky trap/day during the 4-week sampling carried out in Tirana (Albania) in 2011

		Week				Total
		1	2	3	4	
No. of eggs per ovitrap	H_2_O	18.0 ± 32.2	51.4 ± 20.6	59.7 ± 66.9	28.7 ± 34.4	6,312
	H_2_O + Hinf	62.3 ± 74.3	72.0 ± 68.0	98.3 ± 98.3	103.4 ± 107.2	13,209
No. of females per sticky trap	H_2_O	2.1 ± 2.2	2.1 ± 2.1	2.3 ± 2.3	2.8 ± 2.6	368
	H_2_O + Hinf	2.4 ± 2.5	2.9 ± 3.6	3.5 ± 2.7	2.7 ± 2.4	462

H_2_O: only water; H_2_O + Hinf water + hay-infusion

**Fig. 1:**
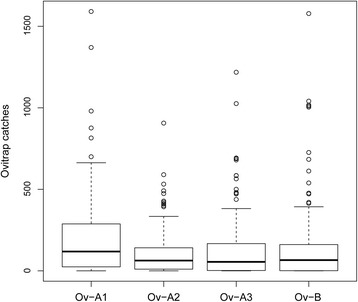
*Aedes albopictus* egg-catches in four ovitraps differing by shape and/or oviposition substrate. Ov-A1: cylindrical ovitrap lined with either heavyweight seed germination paper; Ov-A2: cylindrical ovitrap equipped with a floating white block of polystyrene; Ov-A3: cylindrical ovitrap equipped with a wooden paddle; Ov-B: conical ovitrap equipped with a wooden paddle. The boxes identify the first and third quartiles (the 25th and 75th percentiles). Horizontal black lines within the boxes represent the mean values. The upper whisker extends from the boxes to the highest value that is within 1.5*IQR (inter-quartile range: the distance between the first and third quartiles, so the height of the boxes). The lower whisker extends to the lowest value within 1.5*IQR. Points beyond the end of the whiskers are outliers

Results of the GLMM analysis of the data from Experiment 1 showed that cylindrical ovitraps lined with germination paper yielded significantly higher egg catches than those exploiting as oviposition substrate either the (commonly used) paddles or a floating polystyrene block (Fig. 1, Table 2). Noteworthy, germination paper is already routinely used in the USA [17] and presents several practical advantages over the other oviposition substrates: (i) eggs are evenly distributed and easier to count; (ii) cleaning of the ovitrap internal wall is not required to avoid eggs sticking to these to hatch after the weekly substitution of either paddles or polystyrene blocks; and (iii) dry germination paper sheets can be labelled prior to use and can be easily folded and packed without risking to loose attached eggs during transportation. Results also showed no difference between cylindrical and conical shaped ovitraps (Table 2), which allows to propose the use of conical ovitraps particularly in large-scale monitoring schemes, when transportation of high numbers of traps is needed and the possibility to stack conical ovitraps can be highly convenient. However, it is important to highlight that higher evaporation in conical ovitraps could represent a problem under extreme heat conditions (which were not experienced in Tirana, where weekly mean temperature during the experiments never exceeded 27.3 °C).

**Table 2:** Generalized Linear Mixed Model of *Aedes albopictus* egg catches by different types of ovitraps

Variable	Estimate	Standard Error	*Z*-value	*P*-value
Intercept	4.681	0.459	10.19	< 0.0001
Ov-A2	-0.713	0.145	-4.93	< 0.0001
Ov-A3	-0.791	0.145	-5.45	< 0.0001
Ov-B	-0.696	0.148	-4.69	< 0.0001

For each model coefficient estimates, their standard errors, Wald *Z*-statistic and associated *P*-values are reported. Ov-A1: cylindrical ovitrap lined with either heavyweight seed germination paper (taken as reference level); Ov-A2: cylindrical ovitrap equipped with a floating white block of polystyrene; Ov-A3: cylindrical ovitrap equipped with a wooden paddle; Ov-B: conical ovitrap equipped with a wooden paddle. Number of observations 725; number of weeks = 22

**Fig. 2:**
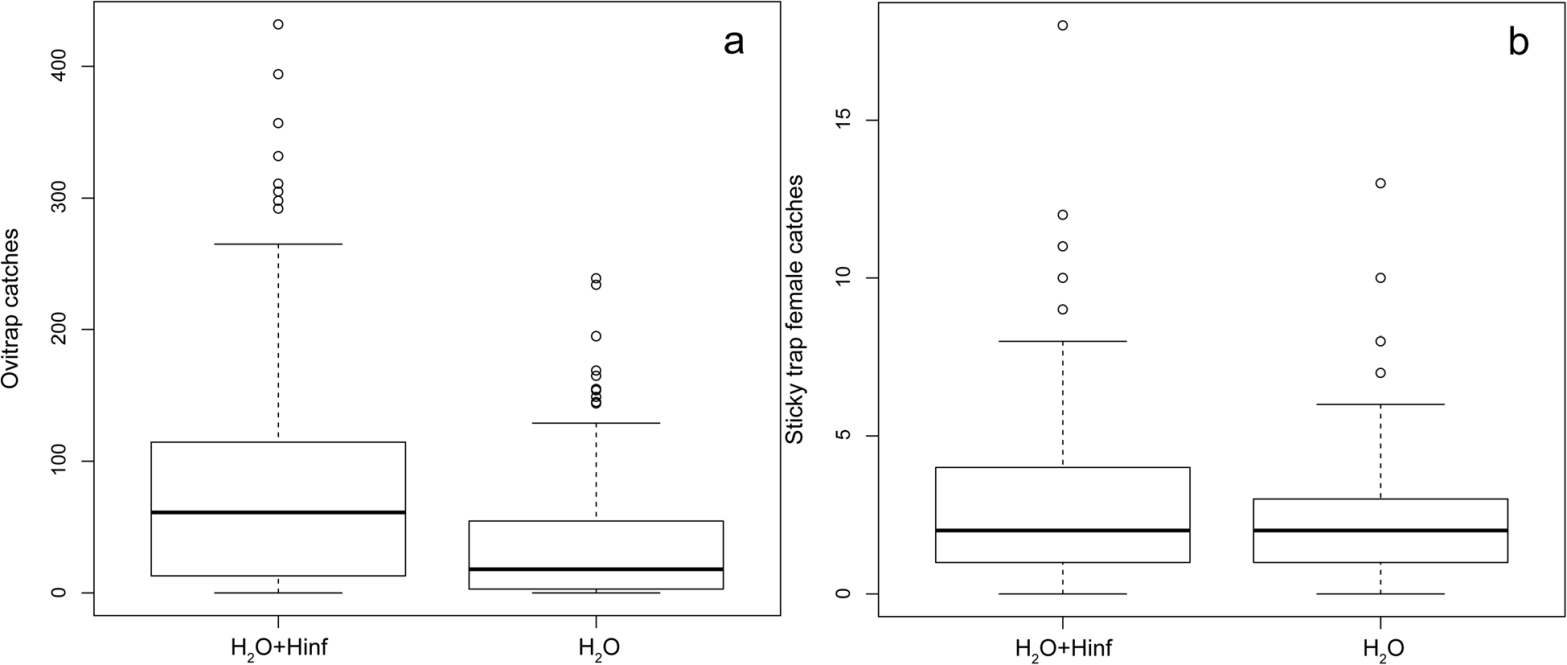
*Aedes albopictus* egg-catches in ovitraps (**a**) and female-catches in sticky traps (**b**) with or without of hay-infusion. H_2_O: water without hay-infusion; H_2_O + Hinf: water + hay-infusion. The boxes identify the first and third quartiles (the 25^th^ and 75^th^ percentiles). Horizontal black lines within the boxes represent the mean values. The upper whisker extends from the boxes to the highest value that is within 1.5*IQR (inter-quartile range: the distance between the first and third quartiles, so the height of the boxes). The lower whisker extends to the lowest value within 1.5*IQR. Points beyond the end of the whiskers are outliers

Results of the GLMM analysis of the data from Experiment 2 showed that ovitraps and STs baited with hay-infusion yielded significantly higher egg and adult female catches than un-baited ones (109 and 26 %, respectively; Fig. 2, Table 3). Although the attractive response of hay-infusion has been demonstrated since several years first for *Ae. aegypti* [18] and later for other mosquito species including *Ae. albopictus* [5–11], the present results show, to the best of our knowledge, for the first time that hay-infusion also increases adult female-catches by sticky traps in the field, an effect so far shown under laboratory conditions only [19].

**Table 3:** GLMMs for numbers of *Aedes albopictus* eggs (response variable for ovitrap model) and adult females (response variable for sticky trap model) collected with or without hay-infusion

GLMM	Variable	Estimate	Standard Error	*Z*-value	*P*-value
Ovitrap model	Intercept	4.262	0.221	16.62	< 0.0001
	H_2_O	-0.863	0.151	-5.72	< 0.0001
Sticky trap model	Intercept	0.966	0.139	6.96	< 0.0001
	H_2_O	-0.227	0.102	-2.22	0.026

For each model coefficient estimates, their standard errors, Wald *Z*-statistic and associated *P*-values are reported. H_2_O + Hinf: water + hay-infusion (taken as reference level); H_2_O: water without hay-infusion. Number of observations = 320; number of weeks = 4

**Fig. 3:**
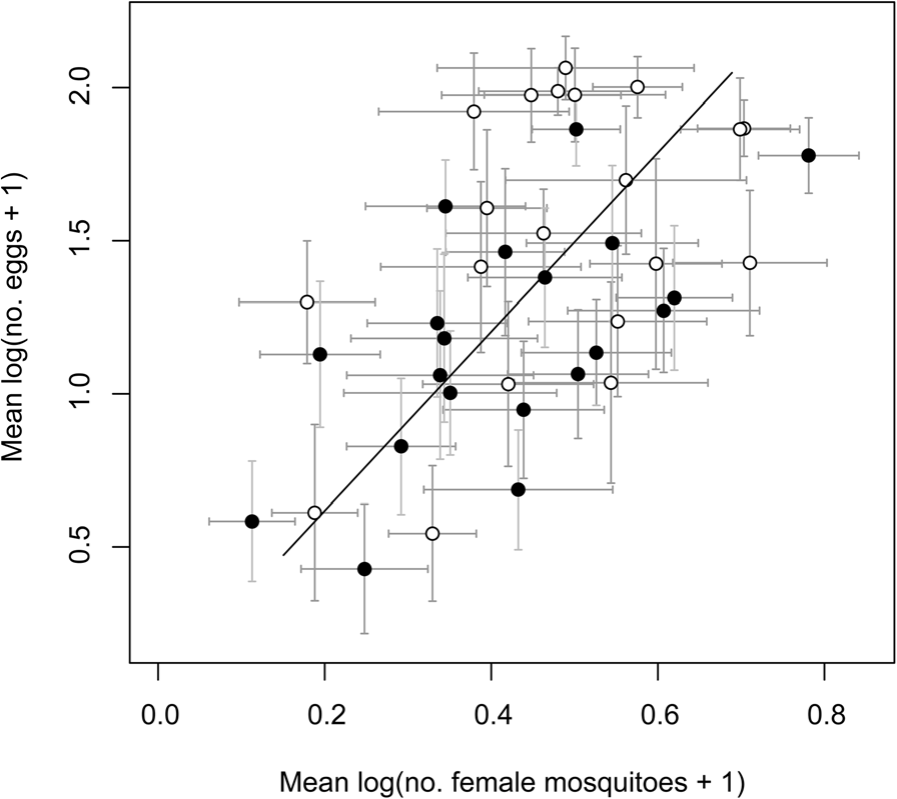
Standardized major axis regression based on means of log-transformed catches of *Aedes albopictus* eggs/ovitrap and females/sticky trap. Filled circles: catches by traps without hay-infusion. Open circles: catches by traps with hay-infusion

A significant correlation between mean egg catches/Ov-A1 and adult female catches/ST was observed over the four week period (Kendall’s Tau = 0.364, *P* < 0.001), although no significant correlation was observed between weekly catches in Ov-A1 and ST (Kendall’s Tau = 0.065, *P* > 0.05), probably due to the high daily variability in trap catches. However, SMA regression revealed a positive relationship between ST and Ov-A1 means of log-transformed catches per site (regression slope on log-scale = 2.92, CI = 2.23–3.83, *P* < 0.001; Fig. 3). The SMA regression did not detect differences in regression slopes between traps with or without hay-infusion (*P* > 0.05), indicating that the significant relationship between ovitrap and ST catches already shown in the absence of attractants [13] is maintained also in the presence of hay-infusion. Moreover, the slope of the SMA regression was > 1, suggesting that, proportionally, Ov-A1 trap catches increase more (around twenty-fold) than ST trap catches at increasing adult female population densities.

Overall, our results provide grounds for optimisation of ovitraps and sticky traps as monitoring tools for *Ae. albopictus*, by (i) supporting the use of germination paper as most appropriate oviposition substrate; (ii) suggesting the possible use of stackable conical ovitraps; and (iii) confirming the use of hay-infusion to increase egg-catches in ovitraps, and showing that this attractiveness also significant increases adult catches by sticky traps.
